# Robotic-Guided Spine Surgery: Implementation of a System in Routine Clinical Practice—An Update

**DOI:** 10.3390/jcm14134463

**Published:** 2025-06-23

**Authors:** Mirza Pojskić, Miriam Bopp, Omar Alwakaa, Christopher Nimsky, Benjamin Saß

**Affiliations:** 1 Department of Neurosurgery, University of Marburg, 35043 Marburg, Germany; bauermi@med.uni-marburg.de (M.B.); omar.wakaa@gmail.com (O.A.); nimsky@med.uni-marburg.de (C.N.); sassb@med.uni-marburg.de (B.S.); 2 Center for Mind, Brain and Behavior (CMBB), 35032 Marburg, Germany

**Keywords:** robotic-guided spine surgery, pedicle screws, intraoperative computer tomography, neuronavigation

## Abstract

**Objective:** The aim of this study is to present the initiation of robotic-guided (RG) spine surgery into routine clinical care at a single center with the use of intraoperative CT (iCT) automatic registration-based navigation. The workflow included iCT with automatic registration, fusion with preoperative imaging, verification of preplanned screw trajectories, RG introduction of K-wires, and the insertion of pedicle screws (PSs), followed by a control iCT scan. **Methods:** All patients who underwent RG implantation of pedicle screws using the Cirq^®^ robotic arm (BrainLab, Munich, Germany) in the thoracolumbar spine at our department were included in the study. The accuracy of the pedicles screws was assessed using the Gertzbein–Robbins scale (GRS). **Results:** In total, 108 patients (60 female, mean age 68.7 ± 11.4 years) in 109 surgeries underwent RG PS placement. Indications included degenerative spinal disorders (*n* = 30 patients), spondylodiscitis (*n* = 24), tumor (*n* = 33), and fracture (*n* = 22), with a mean follow-up period of 7.7 ± 9 months. Thirty-seven cases (33.9%) were performed percutaneously, and all others were performed openly. Thirty-three operations were performed on the thoracic spine, forty-four on the lumbar and lumbosacral spine, thirty on the thoracolumbar, one on the cervicothoracic spine, and one on the thoracolumbosacral spine. The screws were inserted using a fluoroscopic (first 12 operations) or navigated technique (latter operations). The mean operation time was 228.8 ± 106 min, and the mean robotic time was 31.5 ± 18.4 min. The mean time per K-wire was 5.35 ± 3.98 min. The operation time was lower in the percutaneous group, while the robot time did not differ between the two groups. Robot time and the time per K-wire improved over time. Out of 688 screws, 592 were GRS A screws (86.1%), 54 B (7.8%), 22 C (3.2%), 12 D (1.7%), and 8 E (1.2%). Seven screws were revised intraoperatively, and after revision, all were GRS A. E screws were either revised or removed. In the case of D screws, screws located at the end of the construct were revised, while so-called in-out-in screws in the middle of the construct were not revised. **Conclusions:** Brainlab’s Cirq^®^ Robotic Alignment Module feature enables placement of pedicle screws in the thoracolumbar spine with high accuracy. A learning curve is shown through improvements in robotic time and time per K-wire.

## 1. Introduction

The implantation of pedicle screws (PSs) is of central importance for the treatment of spinal instability and the achievement of spinal fusion in degenerative, oncological, infectious, and traumatic diseases of the spine [1]. Standard techniques for PS implantation include freehand implantation, which is always performed as a part of open spine surgery and is based exclusively on knowledge of anatomical landmarks. Historically, this was followed by fluoroscopy-guided (FG) techniques, using intraoperative X-rays (C-arm). The development of cranial navigation in the 1990s subsequently led to the development of navigated PS placement, either using surface-based registration or automatic registration based on intraoperative imaging. The first system for robotic-guided (RG) PS implantation approved by United States (US) Food and Drug Administration (FDA) was SpineAssist by Mazor Robotics in 2004 [1,2]. Over the past two decades, numerous robotic systems for RG PS placement have been introduced to the market. Currently, robotic assistance is primarily used for PS placement [1].The main advantage of RG PS placement is its higher accuracy compared to other techniques, even compared to navigation, as well as the ability to place PSs with larger diameters and longer lengths compared to navigation alone [3].

Despite their advantages, robots involve significant acquisition costs and require extensive training, so there must be compelling reasons for their introduction into standard practice [1]. Our initial experience with RG PS placement in our department, during the first 13 operations and the implantation of the first 70 screws, has already been published [4]. The aim of this study is to describe our experience with the integration of robotic-guided (RG) spine surgery in a single center using intraoperative CT (iCT) navigation based on automatic registration into daily spine care.

## 2. Materials and Methods

All patients who underwent RGimplantation of PSs in the thoracolumbar spine using the Cirq^®^ robotic arm from BrainLab (BrainLab, Munich, Germany) in our department were included in the study. The study is a retrospective evaluation of prospectively collected data. The indication for using a robotic arm included spine instability in degenerative spinal diseases, spondylodiscitis, spinal tumors, and fractures. The robotic arm was used always in combination with iCT for automatic registration. Our department has two operating rooms, one of which is equipped with AIRO^®^ iCT. In addition, the robotic arm could only be used for one operation per day, as only one set of instruments was available for this procedure, including tracking array for the kinematic unit and snap-on depth control. This meant that if two navigated spine surgeries were performed on the same day, the procedure with longer constructs was performed with the robotic arm, and the other with iCT-based navigation and a navigated drill guide. In addition, during the same period, some of the stabilizing spine surgeries were performed using fluoroscopy, usually when the operating room with AIRO-iCT was being used for cranial navigation, such as navigated tumor resection or deep brain stimulation. PS implantation was used in cases with no previous surgery in the region of the spine intended for instrumentation. The open technique was used either in cases with previous surgery in the region intended for instrumentation (e.g., after laminectomy for metastatic epidural spinal cord compression (MESCC) as an urgent surgery, with subsequent stabilization as a secondary procedure), or in cases where additional surgical steps were required in addition to screw implantation, such as decompression of the spinal canal, discectomy, cage implantation, etc.

**Figure 1:**
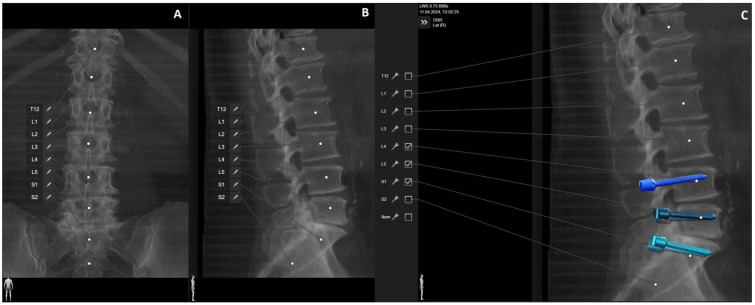
Preoperative (**A**) anterior–posterior (AP) and (**B**) sagittal reconstruction of bone anatomy according to preoperative CT with automatic identification of vertebras. (**C**) Screw recommendations in selected vertebrae for stabilization of L4-S1.

**Figure 2:**
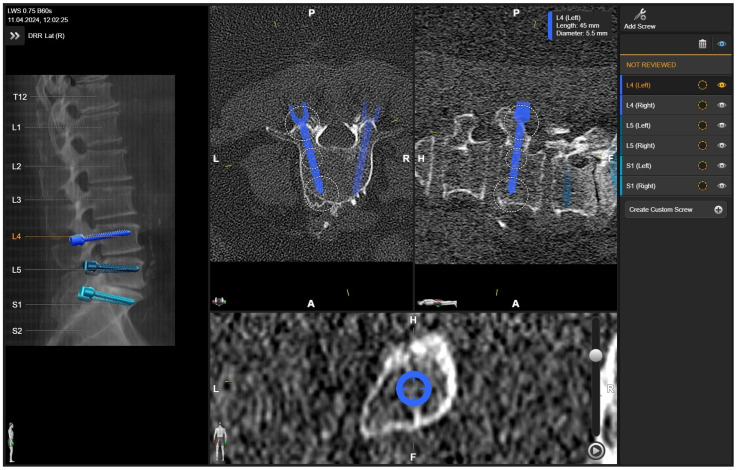
Screw planning includes the entry point, trajectory, diameter, and length of the screw. The proposed screw plan can be modified manually according to the surgeon’s preference.

**Figure 3:**
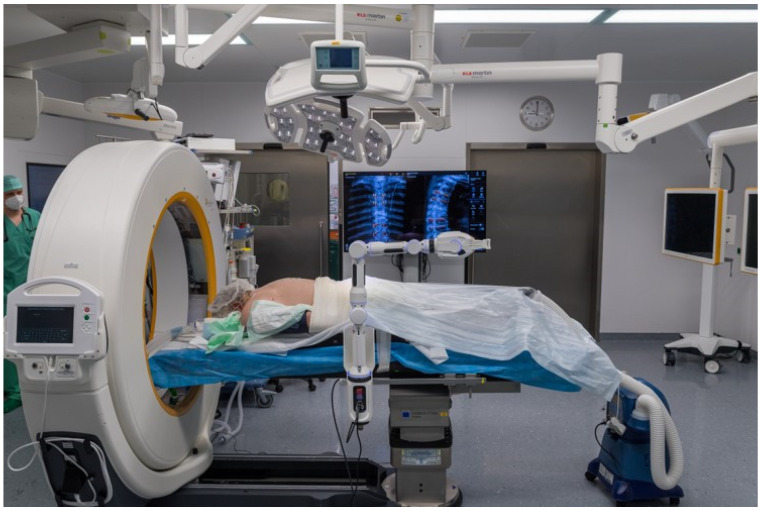
Positioning of the patient.

**Figure 4:**
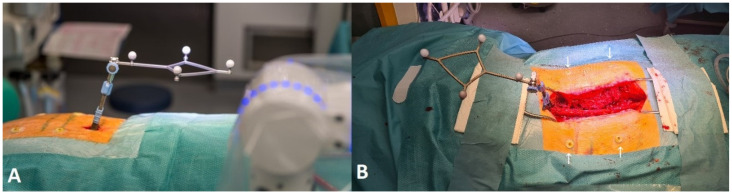
Positioning of the reference array. (**A**) A case with MISS of the upper thoracic spine, with fixation of the reference array via a separate incision caudally from the area intended for instrumentation. (**B**) A case with open surgery to stabilize the thoracic and lumbar spine, where the reference array attached proximally to the surgical area in a spinous process of the vertebrae adjacent to the highest level of the planned spinal construct. Note the skin fiducials on both sides of the planned and actual skin incision (white arrows).

**Figure 5:**
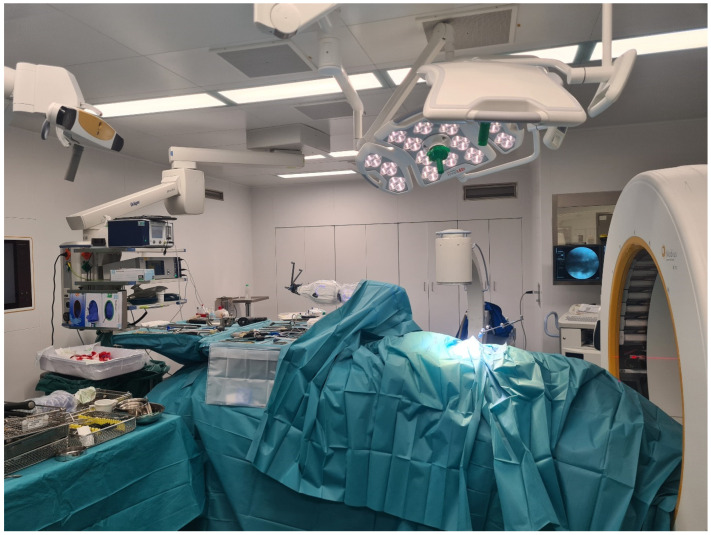
Setting for the registration scan, whereby the surgical site is covered with a sterile drape so that the reference array is visible for the navigation camera.

**Figure 6:**
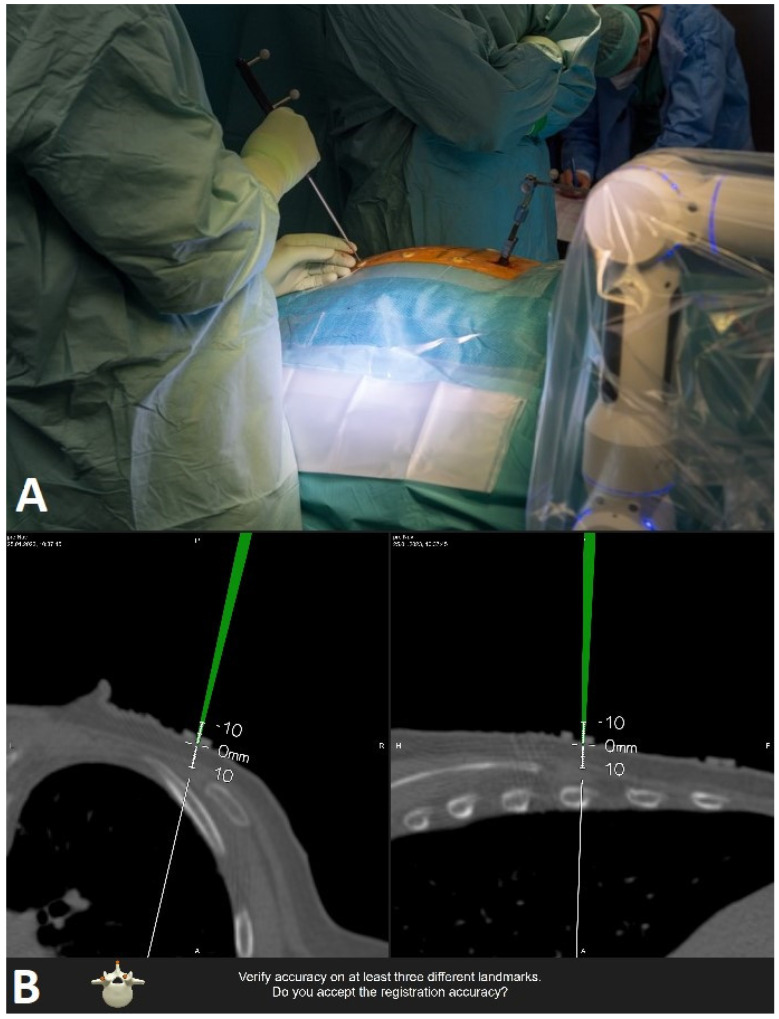
Checking the accuracy of registration on skin fiducials with the pointer. (**A**) position of surgeon with pointer. (**B**) intraoperative screenshot of intraoperative scan in axial and sagittal plane.

**Figure 7:**
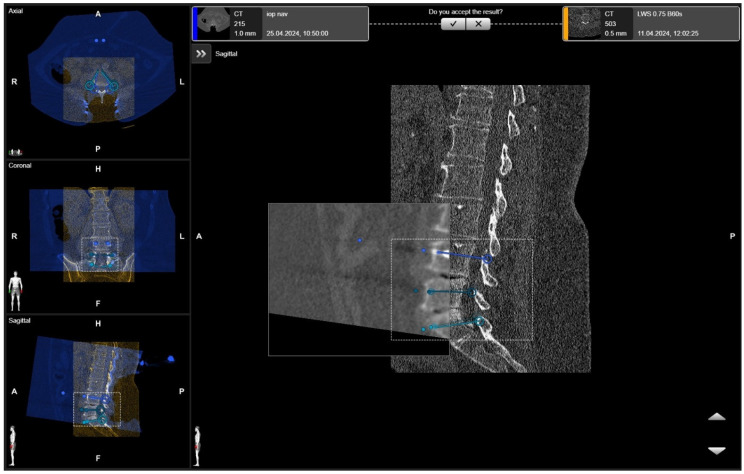
Rigid fusion of selected ROIs of two planned segments for stabilization (same patient as in Figure 1 and Figure 2).

**Figure 8:**
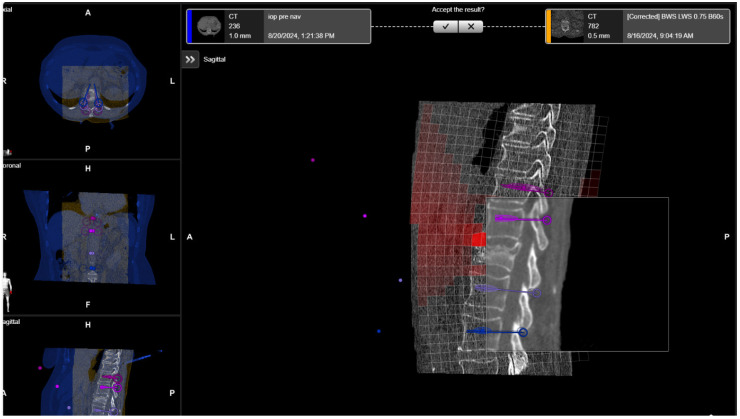
Elastic fusion of the preoperative CT and the iCT scan with preplanned screw trajectories in a fracture at the thoracic–lumbar junction, performed due to instability.

**Figure 9:**
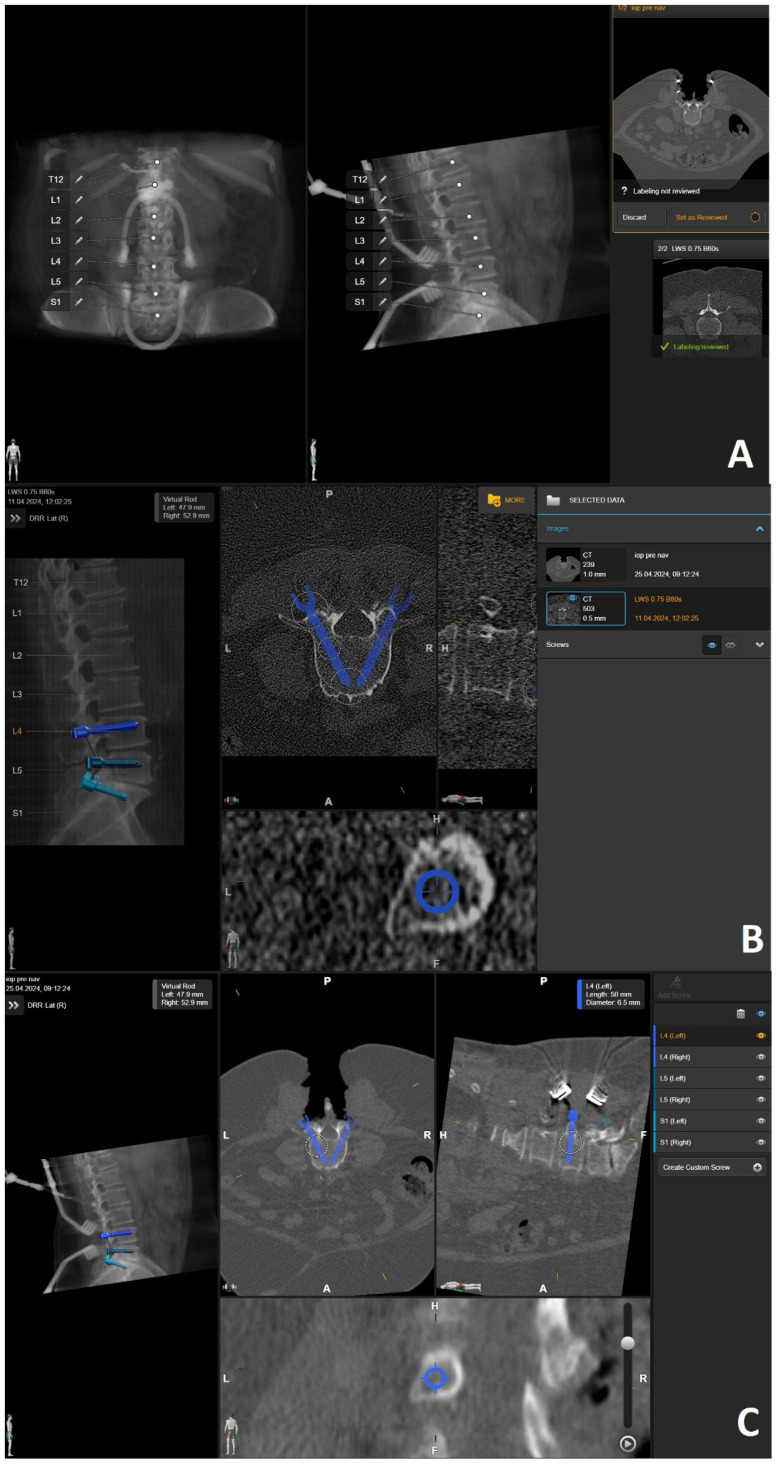
Same patient as in Figure 1, Figure 2 and Figure 7. (**A**) iCT scan with AI-assisted vertebra recognition. (**B**) Screw trajectory in the preoperative CT. (**C**) iCT scan after fusion with preoperative CT shows the screw trajectories, with the option of correcting the screw planning according to the iCT scan.

**Figure 10:**
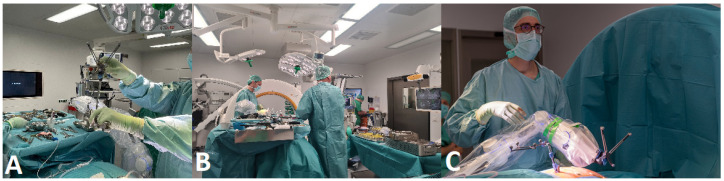
(**A**) Calibration of the tracking array of the robotic arm. (**B**) Position of the surgeon at the OR table, with the robotic arm on the left side and a clear view of the monitor for an overview of the surgical steps. (**C**) Position of the surgeon while using the robotic arm.

**Figure 11:**
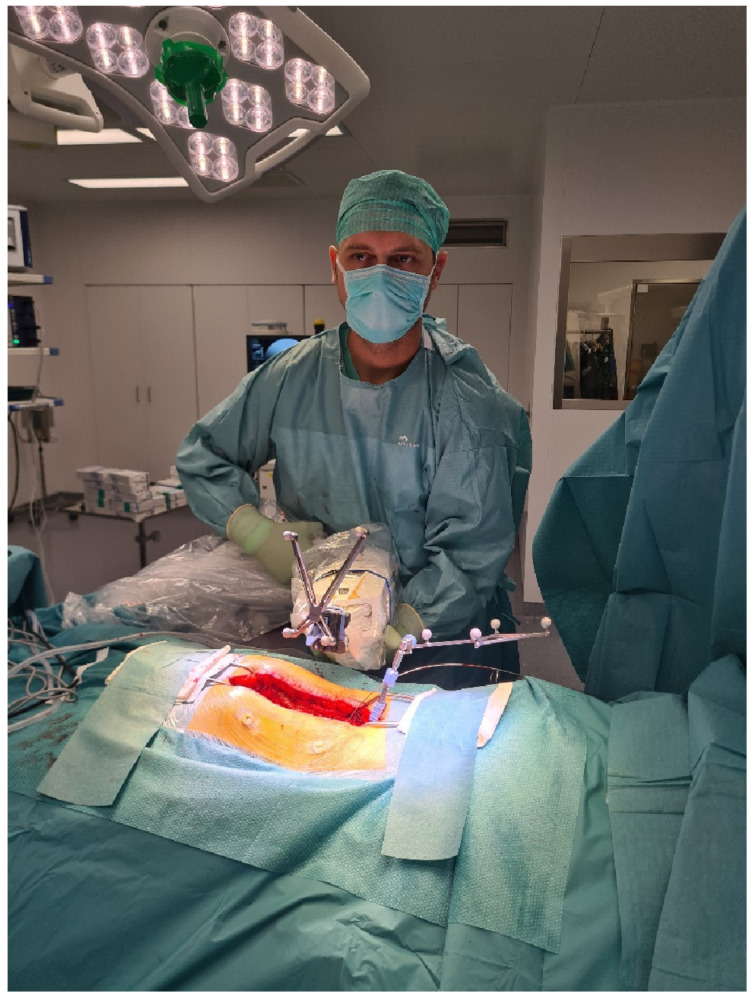
Position of the surgeon with the robotic arm during positioning of the kinematic unit over the projection of the entry point.

**Figure 12:**
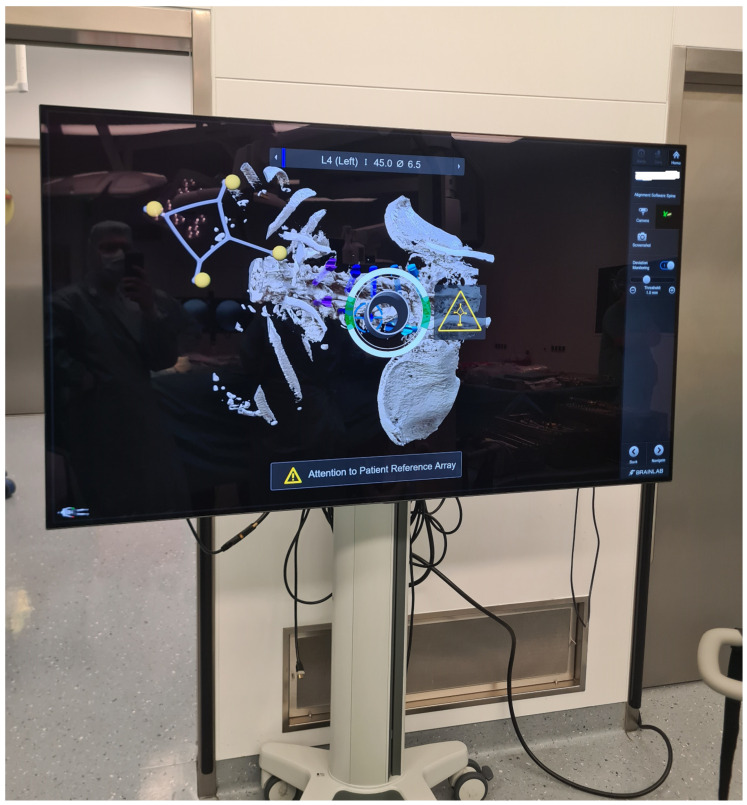
Representation of the bone anatomy and position of the robotic arm projection in relation to the projection of the entry point of the preplanned screw trajectory.

**Figure 13:**
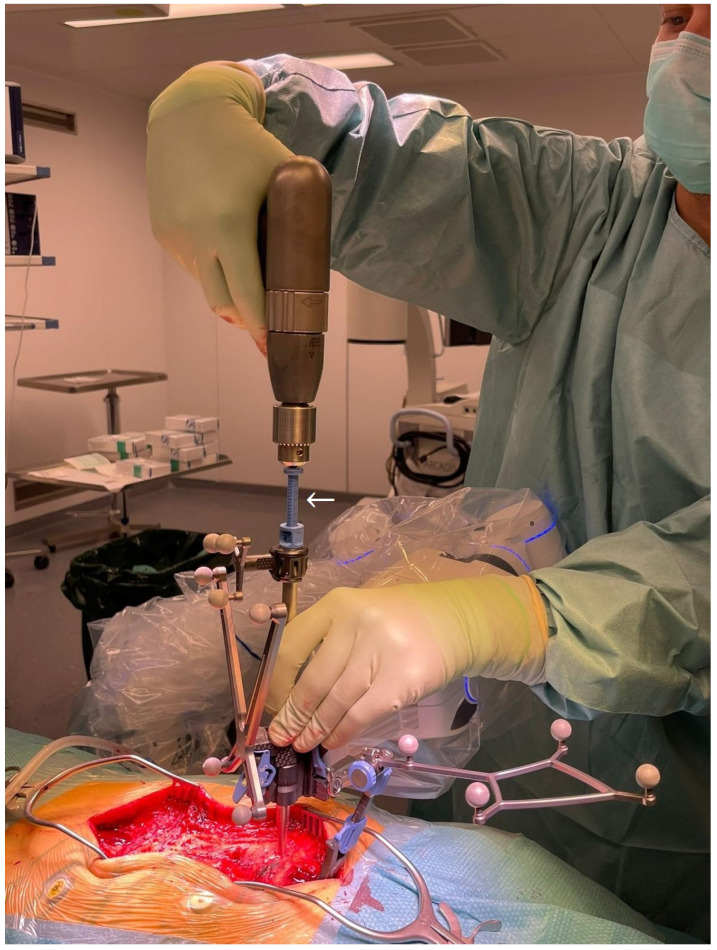
The position of the surgeon with the robotic arm with the tracking array, fixed in position with mounted snap-on depth control (white arrow).

**Figure 14:**
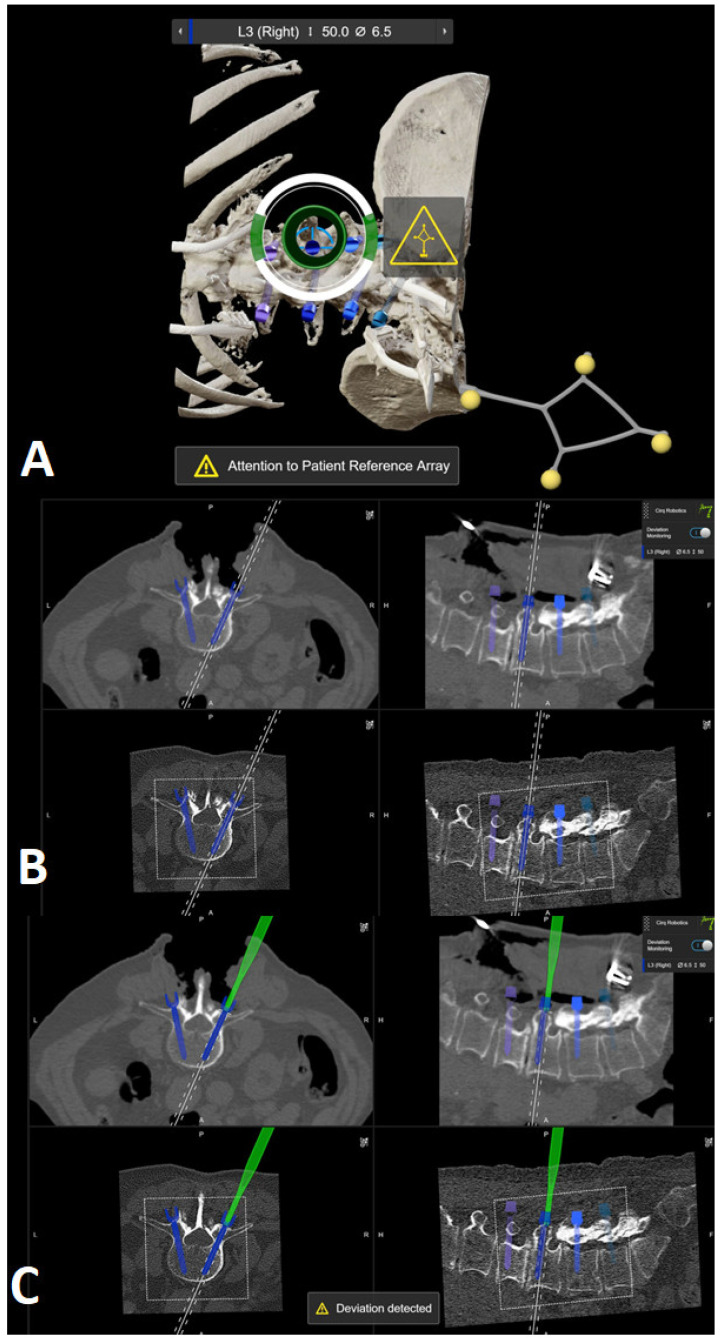
(**A**) Position of the robotic arm when the rough projection of the entry point of preplanned screw trajectory (in this case, the right L3 screw) is reached. The circle representing the kinematic unit of robotic arm turns green, and then the surgeon can release the robotic arm, which then automatically aligns itself according to the preplanned trajectory. (**B**) End position of the trajectory along the preplanned screw in the axial and sagittal view of iCT and preoperative CT. (**C**) Position of the drill guide instrument holder with a tracking array of the robotic arm, which can be securely locked after correct placement at the entry point. The system is very sensitive, so deviations from the original plan are reported very frequently, although these deviations are in the range of 1–2 mm.

**Figure 15:**
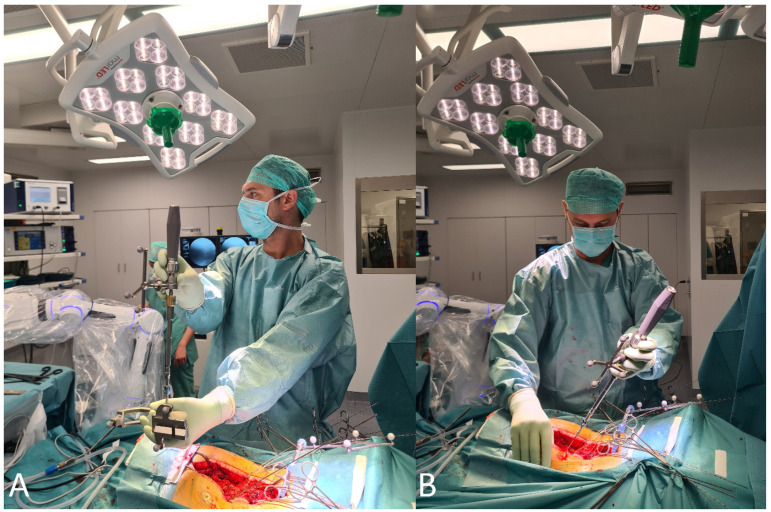
(**A**) Screw calibration and implantation of the (**B**) screw using navigation technology after implantation of all K-wires.

**Figure 16:**
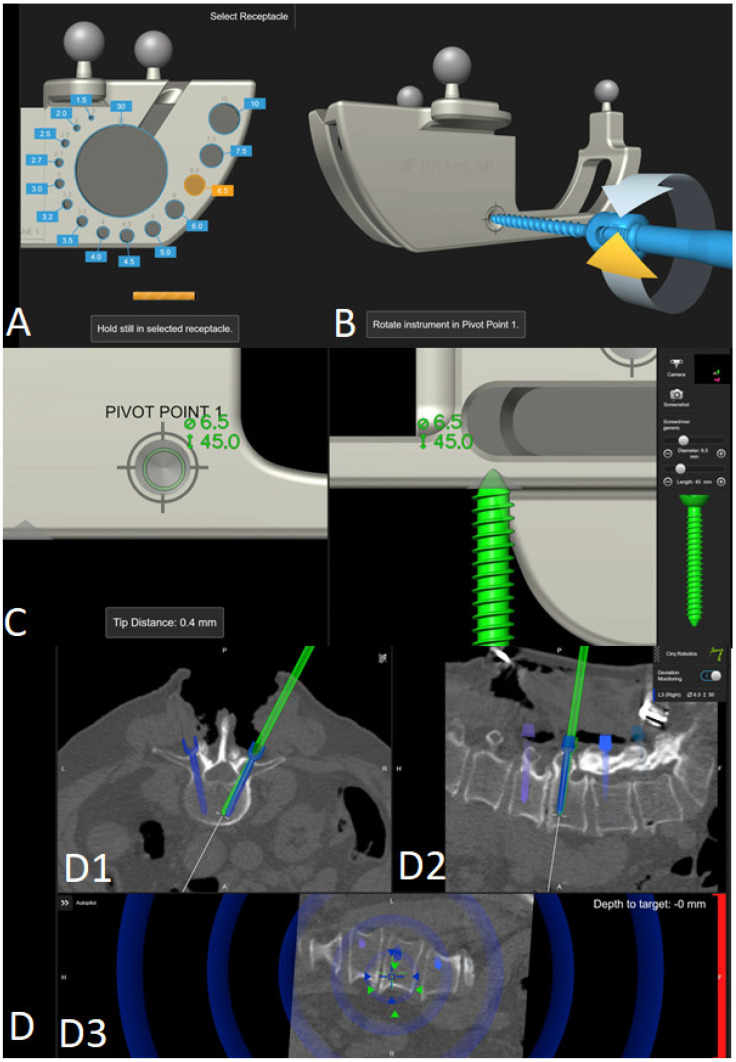
Steps for calibrating the screw, positioning the screw in (**A**) a receptacle of the calibration matrix according to the screw diameter and at (**B**) a pivot point of the matrix with (**C**) final adjustment of screw length and verification of calibration accuracy, with the screw at the pivot point of the matrix. (**D**) Representation of screw implantation in the navigation matrix with preplanned screws (blue) and actual screws (green) in (**D1**) axial, (**D2**) sagittal, and (**D3**) target/perpendicular view.

**Figure 17:**
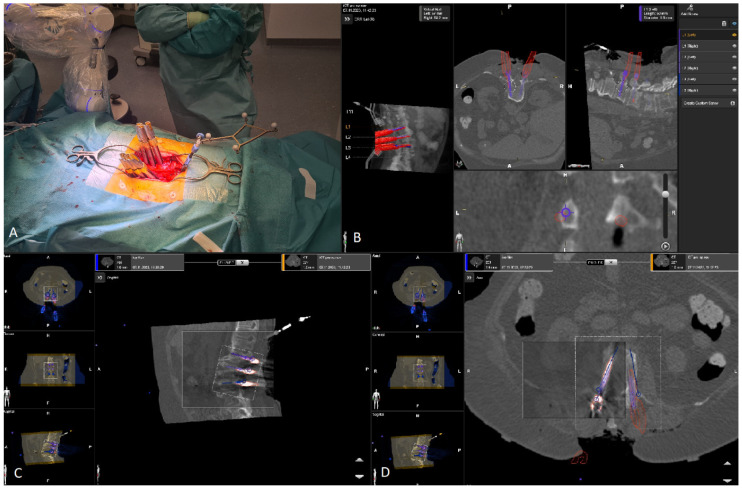
(**A**) Surgical site following stabilization. (**B**) Registration iCT with preplanned trajectories. (**C**) Fusion of registration and control iCT. (**D**) Control iCT with superimposition of preplanned trajectories and actual screws.

**Figure 18:**
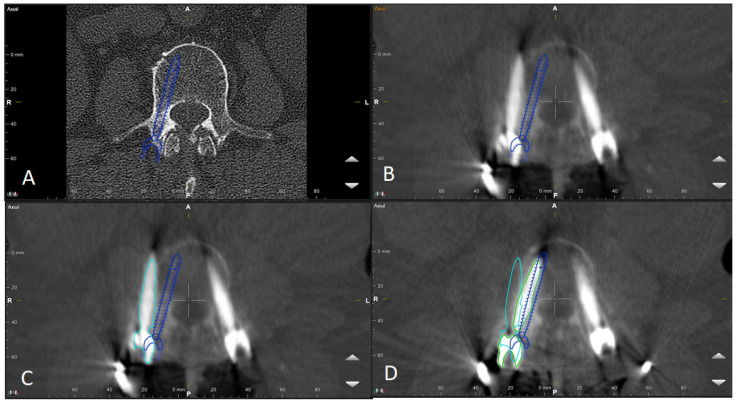
Strategies in visualizing an incorrectly placed screw. (**A**) Visualization of the preplanned screw in the preoperative CT scan. (**B**) iCT to check the screw position shows misplacement of the screw (GRS E) with visualization of preplanned screw. (**C**) Segmentation of the incorrectly placed screw in iCT. (**D**) Control iCT scan with correct position of the screw along the preplanned trajectory and the position of the originally incorrectly placed screw.

Details of operative workflow have already been published [4]. The workflow consisted of the following steps:

1. Preoperative imaging and screw planning. In addition to magnetic resonance imaging (MRI) of the spinal region to be examined and instrumented, all patients received preoperative three-dimensional (3D) CT imaging of the part of the spine that was to be stabilized. The MRI and CT data were exported to the BrainLab navigation software (https://www.brainlab.com/surgery-products/overview-spinal-trauma-products/spinal-navigation/, accessed date 20 June 2025) and fused either rigidly or elastically. The navigation software includes a screw planning application that was used to plan the radius, length, and trajectory of the PS prior to surgery. The software enables automatic identification of the vertebra, followed by automatic screw planning for PSs in the selected vertebra. The proposed preplanned screws were then manually corrected according to the surgeon’s preference. For this step, it was crucial to have an artifact-free, high-resolution 3D CT of the spine, i.e., the area intended for instrumentation. (Figure 1 and Figure 2) [4].

2. The patient is placed in a prone position on a positioning cushion, with additional pressure protection to the knees and elbows, on a carbon fiber surgical table (Figure 3), connected to the AIRO^®^ iCT scanner (Brainlab, Munich, Germany). All anesthesia cables and lines are routed through the gantry. For surgery of the upper and middle thoracic spine, the arms are positioned at the sides of the body; for surgery on the lower thoracic and lumbar spine, the arms are stretched above the head and padded with gel foam paddies and cushions. In all cases, the Cirq^®^ arm is attached to a metal bracket on the left side of the patient. Installation is performed before the patient is covered with sterile drapes [4].

3. The spinal levels intended for instrumentation were determined using fluoroscopy to plan the skin incision. Fluoroscopy was not necessary in previous operations in the same area. Adhesive fiducials (usually four) were applied to the left and right from the planned or existing skin incision to check the accuracy of registration. After this step, the surgical area was prepared and covered, and the Cirq^®^ arm was covered [4].
4. In open surgery cases, the bony structures, laminae, and pedicles were exposed along the area intended for instrumentation. For stabilization of the middle and lower thoracic, lumbar, and sacral spine, a spinous process proximal to the upper vertebra of the planned spinal construct was usually selected for fixation of the carbon reference array. For stabilization of the upper thoracic spine, the reference array was usually placed on the spinous process of the vertebra adjacent to the lowest level of the planned construct. In minimally invasive spine surgery (MISS) involving PS implantation, the reference array was usually attached to the spinous process of the vertebra via a separate small skin incision in the midline with subperiosteal dissection of the muscles of the tip of the spinous process. (Figure 4). It is crucial to position the reference array close enough to the region intended for instrumentation, but also far enough away from the projection of the entry point and screw trajectories of the adjacent instrumented vertebrae to avoid collision of the kinematic unit of the robot arm with the reference array. The skin incision for the percutaneous procedures either a midline incision with exposure of the fascia without opening or multiple skin incisions at the entry points of the screws [4].

5. After exposing the bone anatomy and fixing the reference array, a registration scan with automatic registration of the patient was performed. The surgical area is covered with a sterile drape so that the reference array is visible to the navigation camera, and the scan length is marked with a sterile pen. The registration scan is performed during apnea so that any respiratory artifacts that may occur do not affect accuracy. The scan takes 7–12 s. All personnel leave the operative room for the scan, so that the radiation exposure for staff is zero. Low-dose protocols were used for the scans. The effective dose is calculated by multiplying the total dose length product (DLP) by ED/DLP conversion factors (17.8 μSv/Gy × cm for thoracic and 19.8 μSv/Gy × cm for lumbar spine scans). After the scan, the image data was automatically transferred to the navigation system (BrainLab, Munich, Germany) without user interaction for automatic patient registration. The entire process of covering the patient with a sterile drape for the scan, as well as the scan itself, takes 10–15 min. The registration accuracy was checked and recorded using a pointer and skin fiducials and by placing the pointer in the fixation area of the registration array. In open cases, additional verification of the bony structures was performed. (Figure 5 and Figure 6).

6. After selecting a region of interest (ROI), the iCT scan was merged with a preoperative scan. A rigid fusion was performed, and in cases of instability of the spine due to a tumor or fracture, as well as in cases of longer constructs, elastic fusion was performed (Figure 7 and Figure 8). In selected cases with longer thoracolumbosacral constructs, two scans were performed, with repositioning of the reference array to avoid compromising accuracy for levels far from the reference array. After fusion of the scans, the planned screw trajectories were checked and corrected as necessary (Figure 9) [4].

7. The kinematic unit of the Cirq^®^ Robotic Alignment Module with the previously calibrated tracking array is positioned over the projection of the screw entry point. The instruments are tracked in real time, and their position is constantly reported back so that the surgeon can observe the movements of the robotic arm and all instruments on a separate monitor. The surgeon is located on the side of the robotic arm, i.e., on the left side of the patient. After positioning the robotic arm over the projection of the trajectory entry point, the robotic arm automatically aligns itself according to preplanned trajectory of the relevant screw. In percutaneous cases, a previously calibrated and registered trocar can be used to open the soft tissue for screw implantation, followed by the attachment of an instrument holder for the drill guide. A drill guide is then inserted through the tracking array with the instrument holder and positioned at the entry point, and attachable snap-on depth control for drilling is then attached. The tracking array is then locked, and drilling begins. In this step, it is crucial that the surgeon additionally stabilizes the instrument holder with sufficient pressure on the entry point to prevent skiving. A K-wire is implanted, and the robotic arm is removed. This workflow is repeated for all trajectories. All K-wires are secured to the cover with clamps to keep them in place. (Figure 10, Figure 11, Figure 12, Figure 13 and Figure 14) [4].

8. The screws were inserted using a fluoroscopic (first 12 operations) or navigated technique (subsequent surgeries) technique. During screw implantation, micromovements in the surgical field due to screw implantation can impair navigation accuracy, so screws were first implanted where misplacement could cause the most damage (e.g., thoracic screws vs. lumbar screws or thin pedicles vs. thick pedicles). In all cases, particular care must be taken to avoid collisions and disturbances of the reference array (Figure 15 and Figure 16) [4].

9. After placing the PSs, the surgical site was covered so that the reference array was visible for the navigation camera, and an iCT scan was performed to check the screw position (Figure 17). For longer constructs and spinal instability, the reference array for the second scan was repositioned so that the new scan could serve as both a control iCT scan for implanted screws and a registration scan for screws to be implanted. The second iCT scan is fused with the first registration scan to check the accuracy of screw placements and verify the deviation of the actual screws from the planned trajectories [4]. If the screws are misplaced, the misaligned screws can be segmented into the software to improve visualization after removal and implantation of new screws using the navigated technique. Repeated iCT scans show the visualization of the position of misplaced, repositioned, and corrected screws (Figure 18).

10. In cases where additional surgical steps were necessary, such as decompression of the spinal canal, tumor resection, or implantation of a cage, the reference array was removed and the operation continued. After the completion of these surgical steps, the rods were implanted, and the wound was closed in layers.

Clinical parameters, including diagnosis, neurological deficits, pain, complications, and neurological outcomes, were analyzed based on patient history. Osteoporosis was defined as a T-value ˂ 2.5 in patients who underwent a dual-energy X-ray absorptiometry (DXA) test or as Hounsfield unit (HU) cut-off under 140 at Th12 [5]. The operation time was defined as the time between the first incision and closure. The total time the robotic arm was in use, i.e., the time required for the implantation of all K-wires, was defined as the robotic time, and refers to the period from the positioning of the kinematic unit over the projection of the entry point of the first planned screw trajectory to the implantation of the last K-wire. The time required for the implantation of a single screw was not measured separately. The time per K-wire was calculated by dividing the robotic time with the number of K-wires placed.

The accuracy of the pedicle screws was measured according to the CT-based Gertzbein and Robbins System (GRS). In this system, screw position is classified into classes A to E, based on the extent of breach of the pedicle cortex. An ideal screw is described as an A screw (complete intrapedicular position without penetration of the pedicle cortex), followed by B (penetration of the pedicle cortex < 2 mm), C (2–4 mm), D (4–6 mm), and E (>6 mm or extends further outside of the pedicle). The classification was performed by the first and third authors, with the senior author resolving any discrepancies.

Statistical analyses were performed using the SPSS statistical software, version 20 (SPSS Inc. IBM, 1 Orchard Rd, Armonk, NY, USA), with a *p* value of <0.05 considered to be statistically significant. Kolmogorov–Smirnov and Shapiro–Wilk tests were used to check if variables were normally distributed. Means and standard deviations (SDs) were calculated, and for differences between the means, a *t*-test was used, whereas in measuring differences between standard deviations, Leven’s Test for Equality of variances was performed prior to the *t*-test. In cases where a statistically significant difference between the SDs was found, a *t*-test was not performed. For the comparison of different mean values, an independent sample *t*-test was performed. For the determination of statistical differences between sets of categorical data (for example, between the screw malposition rate in open vs. percutaneous techniques), a Pearson chi-square test was used.

## 3. Results

### 3.1. General Characteristics of the Patients

In total, 108 patients (60 female, mean age 68.7 ± 11.4 years) underwent RG PS implantation, amounting to 109 surgeries at our department during March 2021–December 2024. Forty-seven operations were performed by the first author (43.1%), forty-eight by the senior author (44%), eleven by the first and senior author (10.1%), and three by two other treating surgeons.

Indications included degenerative spinal disorders (*n* = 30 cases), spondylodiscitis (*n* = 24), tumors (*n* = 33), and fractures (*n* = 22), with a mean follow up of 7.7 ± 9 months. There were no significant differences in mean age, gender, and BMI between the four indication groups (*p* > 0.05). All degenerative cases were transforaminal lumbar interbody fusion (TLIF) cases, performed on the lumbar and lumbosacral spine.

Patients with infection presented with spondylodiscitis with or without intraspinal empyema in the thoracic and lumbosacral spine. One patient with spondylodiscitis in thoracic and lumbar spine underwent two separate RG surgeries for stabilization. Patients with fractures due to trauma had osteoporosis more frequently (*t*-test, *p* < 0.05) than other patient groups. Fractures were localized in the lumbar spine in four cases, in the thoracic spine in one case, and at the thoracic–lumbar junction in 17 cases. In 19 cases, stabilization with fracture reduction was performed, and in three additional cases, decompression of the spinal canal was performed. Osteoporosis was present in 38 cases (34.9%). Of these patients, only 14 patients received treatment for osteoporosis at the time of surgery.

In patients with a tumor, two cases involved primary spinal tumors (chondrosarcoma and giant cell tumor), 28 cases involved metastases with instability and/or pathological fracture, two cases involved multiple myeloma of the spine, and one case involved a neurinoma with bone erosion and instability of the spine. The most common primary tumor was breast cancer (ten cases), followed by metastases from lung cancer (nine cases) and prostate cancer (five cases), as well as one case each of hepatocellular carcinoma and adenocarcinoma of the stomach, esophagus, and bladder.

In total, 34 patients (31.5%) had motor neurological deficits prior to surgery. Of the 108 patients, 9 improved, 2 deteriorated, and 97 remained unchanged following surgery.

### 3.2. Mortality

Thirteen patients (12%) died during follow-up, ten with metastases, two with spondylodiscitis, and one with fracture. Of these, four patients died during the hospital stay, within less than 30 days following surgery (two patients with spondylodiscitis due to septic shock, one patient due to the progression of metastatic disease with multiple spinal and intracranial metastases, and one patient with metastatic spine disease due to fulminant pulmonary embolism on the second day after surgery). Of the nine other patients, one patient with a fracture died 7 months following surgery and two patients with spondylodiscitis died one year following surgery for reasons unrelated to surgery and disease. Six patients with tumors (two cases each of breast and lung cancer, multiple myeloma, and prostate cancer) died due to the progression of the tumor disease until the end of the follow-up. The mortality rate was higher in tumor and infection cases than in degenerative and trauma cases (*t*-test, *p* < 0.05).

### 3.3. Invasiveness of Surgery

A total of 37 cases (33.9%) were performed percutaneously, with the rest using an open technique. In all, 33 operations were performed on the thoracic spine, 44 on the lumbar and lumbosacral spine, 30 on the thoracolumbar spine, 1 on the cervicothoracic spine, and 1 on the thoracolumbosacral spine. In nineteen cases, one segment was stabilized; in twenty-seven cases, two segments; in ten cases, three segments; in thirty-four cases, four segments; in twelve cases, five segments; in three cases, six segments; and in four cases, seven segments. The mean number of fused segments was 3.17 ± 1.6.

In 43 cases, screw implantation was the only surgical procedure (21.1%); in the remaining cases, additional surgical steps were necessary, such as decompression of the spinal canal, evacuation of epidural empyema, discectomy and cage implantation, tumor resection, and biopsy. Of the 37 patients who underwent surgery using the percutaneous technique, 36 had previously undergone surgery in the region of the spine intended for instrumentation. Of the 72 cases involving instrumentation using the open technique, 17 patients had previous surgery on the instrumented segments. These patients underwent either extension of the spinal construct due to adjacent segment disease (three patients) or underwent prior urgent decompression of the spinal canal due to intraspinal empyema or MESCC (fourteen patients) and then subsequent RG stabilization.

### 3.4. Operative Time, Robotic Time and Time-per-K-Wire

The mean duration of surgery was 228.8 ± 106 min and the mean robotic time was 31.5 ± 18.4 min (*n* = 109). In 43 cases, only stabilization was performed; in the remaining cases, additional procedures included decompression of the spinal canal, tumor resection in oncological cases, cage implantation, biopsy, or expandable cage implantation. In cases where only stabilization was performed, the mean operative time was 177.7 ± 72.1 min (*n* = 43), and in rest of the cases, it was 263.4 ± 109.7 min (*n* = 66), a difference that was significant (*p* < 0.05). The mean time per K-wire was 5.35 ± 3.98 min. The mean time per K-wire was shorter in the last 54 cases (4.53 ± 1.43 min) than in the first 55 cases (6.16 ± 5.3 min), although this was not statistically significant (*p* > 0.05). The mean time per K-wire (08.27 ± 06.54 vs. 4.63 ± 1.52) and the mean robotic time (28.64 ± 11 vs. 45.40 ± 34.29) were significantly shorter in the last 97 cases than in the first 12 cases (*p* < 0.05). The operation time was shorter in the percutaneous group (162 ± 62.8 min percutaneous vs. 277 ± 369.5 with the open technique, *p* < 0.05), while the robotic time did not significantly differ between the two groups (*p* > 0.05). There were no differences in operative and robotic time between two surgeons with the highest number of cases, as well as between the different indication groups (*p* > 0.05).

### 3.5. PS Accuracy

A total of 688 screws were implanted in the RG technique, with a mean of 6.31 ± 2.2 screws per case. Out of 688 screws, 565 were GRS A screws (82.1%), 54 B (7.8%), 22 C (3.2%), 12 D (1.7%), and 8 E (1.2%). Twenty D and E screws were used in eighteen patients. Seven screws were revised intraoperatively (in chronological order—two D screws in case number 6, one D screw each in patient 29 and 79, and one E screw each in cases 42, 60, and 66), and after revision, all were GRS A. Of the eight E screws, three were revised and five removed. Of the twelve D screws, four screws, which were at the end of the construct, were revised, and ten so-called in-out-in screws in the middle of the construct were not revised. Therefore, the rate of suboptimal, clinically unacceptable screws was 2.9%, with an intraoperative revision rate of 1%. There were no neurological deficits resulting due to incorrectly placed screws and no vascular injuries. There were no differences in the rate of GRS A and B screws or in the rate of incorrectly placed screws between the two surgeons with the highest number of cases and between the regions of the spine (*p* > 0.05).

### 3.6. Robot Abandonment

In nine cases, the robotic arm was abandoned, so a navigated drill guide was used instead. In five cases, this was due to ergonomic problems, and in two cases, it was due to technical problems. These screws were not included in the final evaluation. In three cases, RG PS placement was planned, but due to technical problems prior to PS implantation, such as problems with the installation and restart of the system, surgery was performed using the navigated drill guide.

### 3.7. Complications

Complications occurred in 29 patients, including cases with intraoperatively revised and/or removed screws. Surgery in one patient with a lumbar fracture and the L1-S1 construct was aborted following screw implantation due to cardiopulmonary instability but successfully continued the next day. Wound healing disorders occurred in 11 patients, resulting in cerebrospinal fluid (CSF) leaks in two patients. One patient experienced epidural hemorrhage and underwent revision surgery to remove the hematoma. Four patients experienced hardware failure: one patient with rod dislocation, one case with revision of the expandable cage, and one case each with screw pullout and screw loosening. One patient underwent RG surgery for adjacent segment disease, and six patients developed a disease of the adjacent segment during follow-up.

Patients with osteoporosis had a higher incidence of complications, both hardware-related and non-hardware-related (*p* < 0.05). The BMI, choice of operative technique, treating surgeon, and underlying disease showed no correlation with the complication rate, neither for the overall complication rate nor for screw malposition or hardware failure (*p* > 0.05).

### 3.8. Learning Curve

**Figure 19:**
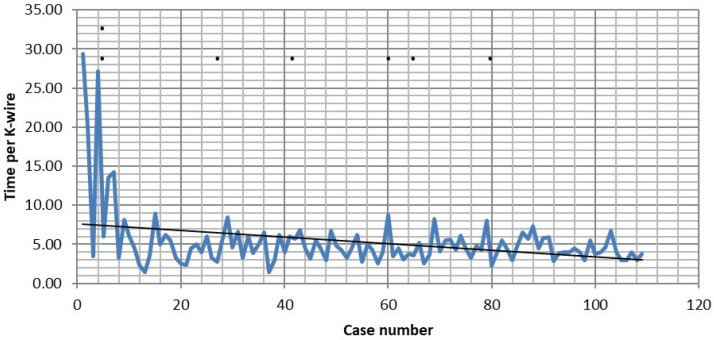
Reduction in time per K-wire during the observation period. Points (•) indicate cases with intraoperative screw revision.

A learning curve is reflected in improvements in robotic time and time per screw throughout the observation period (Figure 19). The frequency of GRS A and B screws, as well as blood loss, screw misplacement, and overall complications, remained unchanged over time.

### 3.9. Radiation Exposure

The C-arm was used in a total of 87 cases to determine the level prior to surgery or for other surgical procedures. The mean dose length product for these patients was 114.4 ± 209.3 mGycm. All patients underwent at least two iCT scans (a registration scan and a scan to check the implant position) using low-dose protocols. In eighty-seven cases, patients received two iCTs. Three or more iCTs were performed in one case of screw revision and cases where a long construct with spinal instability was performed, requiring the reference array to be repositioned with repeated registration scans throughout the operation. In ninteen cases, three iCTs were performed; in two cases, four; and in one case, five scans. The mean total effective dose was 11.05 ± 6 mSv.

### 3.10. Illustrative Cases

#### 3.10.1. Case 1

**Figure 20:**
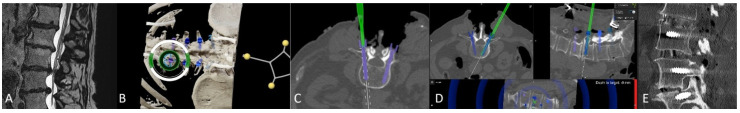
(**A**) Preoperative T2-weighted MRI of the lumbar spine shows spinal canal stenosis and instability. (**B**) Intraoperative screenshot after positioning the kinematic unit of the robotic arm over the preplanned trajectory. (**C**) Position of the instrument holder at the entry point. (**D**) Intraoperative screenshot during screw implantation. All screws were GRS A. (**E**) Sagittal postoperative CT with screws and cages.

A 76-year-old patient with instability of the lumbar spine and a facet joint cyst at L3/4, following previous decompression at L2/3 on the right and L4/5 on the left, underwent RG PS implantation at L2–L5 with cage implantation (Figure 20).

#### 3.10.2. Case 2

**Figure 21:**
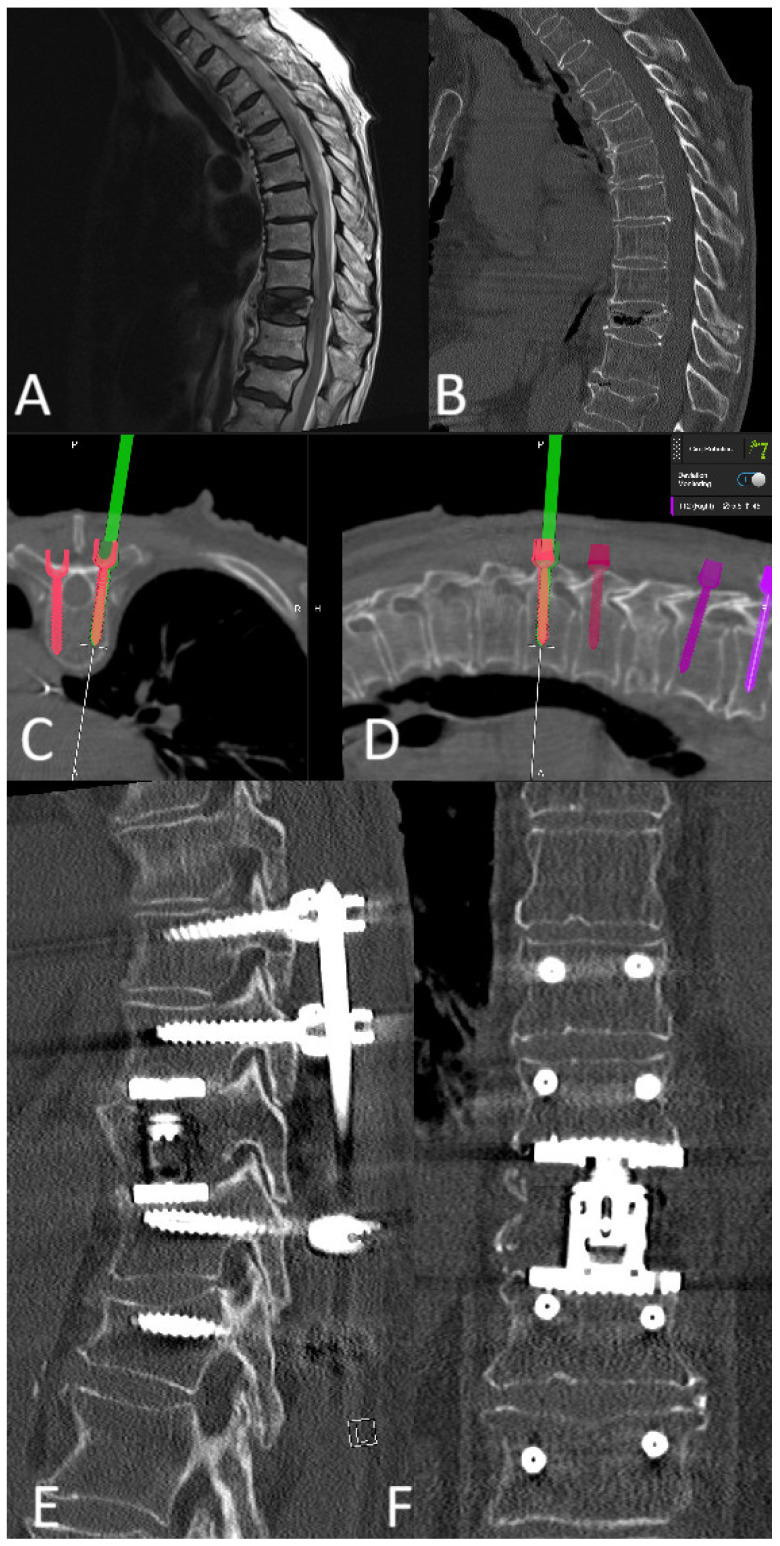
Preoperative (**A**) T2-weighted MRI and (**B**) CT of the thoracic spine, showing a pathological fracture of Th10 with instability. The patient underwent RG placement of K-wires followed by navigated placement of PS, shown here in intraoperative (**C**) axial and (**D**) sagittal view. In addition, an expandable vertebral body cage was implanted via a left transthoracic approach. Postoperative CT in (**E**) sagittal and (**F**) coronal view.

A 78-year-old patient with a pathological fracture of Th10 due to breast cancer metastasis underwent multi-stage surgery: RG PS placement at Th8/9-L1/2, followed by implantation of an expandable vertebral body cage via a left transthoracic approach. All screws were GRS A (Figure 21).

#### 3.10.3. Case 3

**Figure 22:**
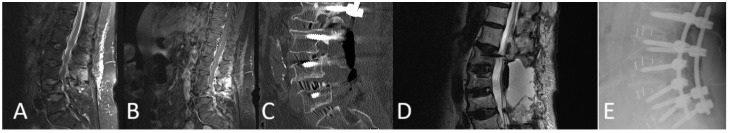
Preoperative sagittal MRI: (**A**) T2-weighted and (**B**) T1 post-contrast MRI shows an empyema with spondylodiscitis and a fracture of the L4 vertebra. The patient underwent RG PS placement at L2-S1, followed by cage implantation. Nine screws were GRS A, and one screw was GRS C. Postoperative sagittal lumbar spine (**C**) CT, (**D**) T2-weighted MRI, and (**E**) X-ray.

**Figure 23:**
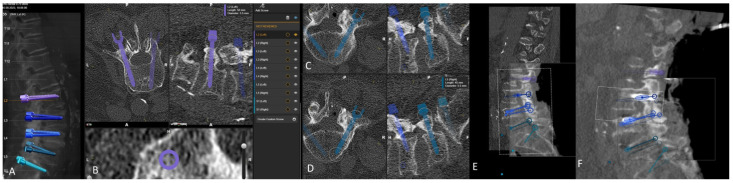
Preoperative planning of PS for stabilization of L2-S1 according to preoperative CT in (**A**) sagittal view after automatic detection of vertebrae and (**B**) planning of the position, length, and diameter of each screw in axial, sagittal, and perpendicular view. (**C**) Axial and sagittal view of the right L5 screw with automatic planning—note that the screw tip is aligned towards the endplate. (**D**) Position of L5 screw right after manual repositioning. (**E**) Fusion of preoperative CT and iCT registration scan. (**F**) Fusion of the registration and control iCT scan.

**Figure 24:**
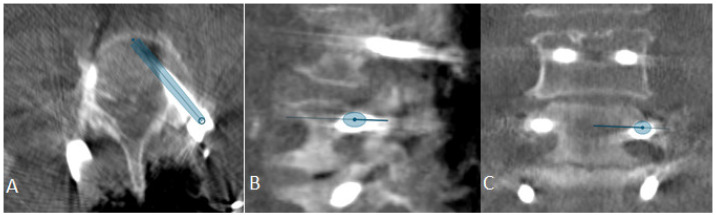
Each screw in the control iCT scan can be viewed in the (**A**) axial, (**B**) sagittal, and (**C**). coronal views, comparing the actual screw position with the preplanned screw trajectory.

An 81-year-old patient with spondylodiscitis, intraspinal empyema at L2-S1, and an L4 fracture underwent decompression for empyema evacuation and RG PS placement at L2-S1, followed by cage implantation (Figure 22, Figure 23 and Figure 24).

#### 3.10.4. Screw Revision Case

**Figure 25:**
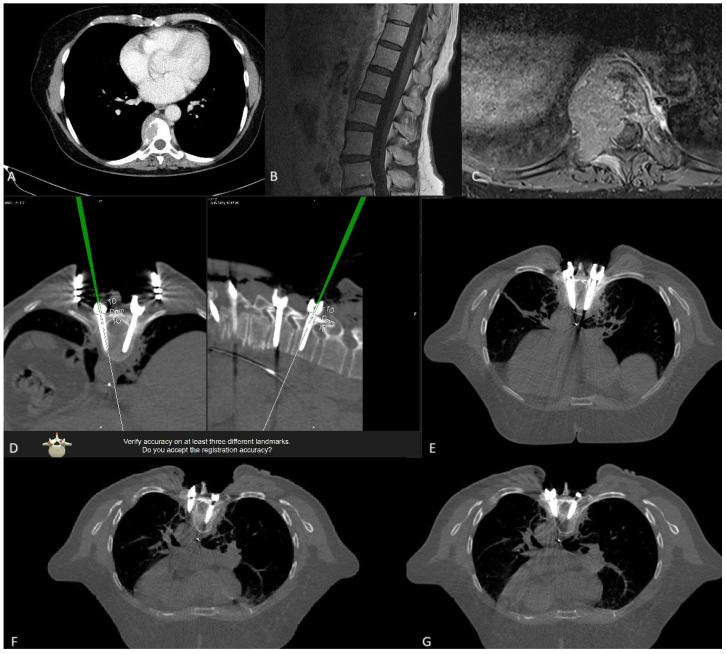
(**A**) The preoperative CT scan six months before surgery shows an osteolytic lesion in the right Th9 pedicle. A CT-guided biopsy was performed in another department, confirming the tumor, whereupon radiation therapy was initiated. Due to progressive pain with spinal ataxia, an MRI scan of the spine was performed. The preoperative T1 post-contrast (**B**), sagittal, and (**C**) axial MRI results show tumor growth from the right pedicle with vertebral body collapse and compression of the dural sac. The patient underwent RG PS placement. The reference array was placed on the spinous process of Th7 and a total of 8 PSs were implanted. (**D**) Accuracy check with a pointer in the iCT scan to check the PS position. (**E**). Th8 GRS A PS. (**F**). iCT shows malposition of the Th7 screw on the left side, a GRS E screw, without any injury to vessels, pleura, or lungs. After removal of the screw, a new RG PS was inserted. (**G**) The correct position, shown in a repeated control iCT scan.

A 63-year-old female patient with Th9 metastasis of renal cell carcinoma underwent Th7/8-10/11 RG stabilization with decompression of the spinal canal and partial resection of the tumor. The reference array was placed proximally to the surgical field and fixed to the spinous process of Th7. K-wires were inserted in a proximal-to-distal sequence, and screws were placed in a distal-to-proximal sequence. The distal screws (Th10/11) were GRS A screws, while the last screw (left Th7 screw) was a GRS E screw due to possible unintended movement of the reference array, which led to major navigation inaccuracy and further screw skiving. Fortunately, no major vessels or pulmonary injuries occurred. The screw was removed, and a new screw was inserted, this time a GRS A screw. (Figure 25).

#### 3.10.5. Robot Abandonment Case

**Figure 26:**
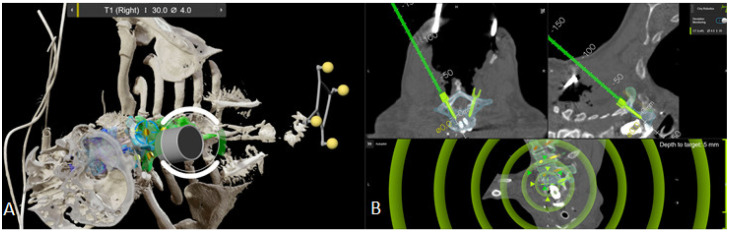
Robot abandonment due to ergonomic problems. (**A**) An intraoperative screenshot shows the failed positioning of the kinematic unit of the robotic arm along the preplanned screw trajectory due to the angle and soft tissue of the patient. (**B**) Th1 right PS placement using navigated drill guide.

A 77-year-old adipose patient with Bechterew’s disease suffered a C6 fracture with instability. He underwent a multi-stage operation, in which the ventral fracture was reduced, and an expandable vertebral body cage with plate was implanted, followed by dorsal stabilization with lateral mass screws at C3/4/5/6 and PSs at C7/Th1/2/3. The reference array was placed on the spinous process of Th5. The cervical screws were placed using the navigated drill guide. Thoracic PSs were planned for the RG surgery. The robotic arm was attached to the left side of the operating table. In the case of the Th1 PS on the right side, the trajectory angle and the soft tissue of the obese patient did not allow correct positioning of the kinematic unit of the robotic arm, so this screw was implanted using the navigated drill guide (Figure 26).

## 4. Discussion

### 4.1. Learning Curve in RG Spine Surgery

The aim of our study was to describe the introduction of RG surgery in a single center by surgeons with no previous experience in spinal robotics. RG surgery demonstrated a short learning curve, with studies showing improved screw accuracy, especially in thoracic spine and deformity cases, and reduced blood loss compared to freehand techniques, even for less experienced surgeons. While some reports note initial improvements in efficacy and time per screw, others suggest minimal or no learning curve with consistent accuracy and no major differences between surgeons with different experience levels [6,7,8,9,10]. A prospective study involving 1120 freehand-placed PSs, the FG and RG techniques showed a steady increase in PS placement accuracy (increased incidence of GRS A and B screws) and less blood loss with the RG technique using intraoperative O-arm imaging [10]. One of the main advantages of the RG technique, as demonstrated in a series of 769 RG procedures compared to 788 freehand PSs by Feng et al. [11], is that the RG technique allows a short learning curve even for junior surgeons, based on a cumulative summation (CUSUM) analysis, and delivers constant good results from the early stages on. In this series, a difference in PS accuracy was shown, with superior results in the upper thoracic region and in cases of deformity compared to the conventional freehand technique [11]. The superiority of the RG technique in thoracic spine surgery compared to the freehand and FG techniques was demonstrated in a meta-analysis of 51,161 PSs, although without differences compared to iCT-based navigation [12]. Siddiqui et al. [8], in an analysis of their first 120 cases with experienced surgeons and fellows, reported increased accuracy following the first 30 screws, with similar adaptation rates for both experienced surgeons and fellows. Interestingly, the screw accuracy rate, i.e., the rate of GRS A and B screws, as well as the rate of misplaced screws over time, did not show a difference in frequency. In addition, we had a unique setting in which two spinal surgeons learned the technique in parallel and immediately passed on the techniques they had learned from the experienced to the less experienced surgeons, which is also the reason for comparable screw accuracy and misplacement rates. Avrumova et al. described improvements in screw time for the first several cases in their series of 311 PSs, with 93.5% of screws fully contained within the pedicle [9]. In an analysis of 33 patients by Urakov et al. [13] a trend toward efficiency was observed. Similarly to our study, there were no differences in time per screw between surgeons with varying levels of involvement in spinal surgery [13]. Minimal learning curves with no differences between surgeons with varying levels of experience were noted by Khan et al. [14] and Vardiman et al. [15]. Robotics has proven useful for less experienced surgeons in deformity surgery in terms of lower deviations and shorter times per screw [16,17]. Higher costs and longer surgery and anesthesia times are disadvantages of RG PS implantation [18]. Kam et al. [19] described their experience with the first 80 cases of RG spine surgery, with a PS revision rate of only 1/352 and a high rate of clinically acceptable screws, but a long time per screw of 7 min and no significant differences in these parameters between initial experience and experience after one year, suggesting that there is a very short or no learning curve. A short learning curve was also demonstrated by Fayed et al. in their first 103 PSs, with a total breach rate of 5.8% and more breaches in the first half of the cohort [20]. Similarly, McCormick et al. [21] reported no misplaced screws and a failure rate of 1.7%. in their first 100 cases and 1056 PSs. Similarly to our study, Jiang et al. also showed a reduction in operative time in 234 cases. [22], with general competence in RG surgery being achieved after the 20th case. Similarly to these results, a mathematical model developed to fit the learning curve in 167 cases with a ROSA^®^ robot by Hsu et al. [23] observed a general learning trend that was attributable to screw planning, with the largest change at the time of the 20th operation. Jiang et al. defined 67 cases as the threshold for achieving mastery [22].

### 4.2. Studies Applying the Cirq^®^ Robotic Arm

The Cirq^®^ Robotic Alignment Module is a unique cost-effective bed-side mounted robotic arm, because it involves exclusively K-wire-guided PS placement. This also makes the system less expensive than other robotic systems available. After K-wire placement, it is possible to place PSs over K-wires without imaging or using a C-arm. This setting is increasingly preferred and recommended among Cirq^®^ users, since some of PS misplacement can be attributed to navigation inaccuracy of the screw driver and navigated PSs. While it reduces surgeon and patient radiation exposure and enables intraoperative verification, its lighter, bed-mounted design limits reach—particularly in the thoracic levels—and may reduce rigidity, potentially increasing misplacement risk. Studies have shown mixed efficiency outcomes, with some reporting faster screw placement but others noting longer operative times and higher complication rates. In addition to our initial experience mentioned above, several studies have demonstrated high PS accuracy with the Cirq^®^ system. The Cirq^®^ used in the current study features “active” assistance, i.e., automatic alignment of the kinematic unit according to the preplanned screw trajectory [4]. Analysis of 25,054 screws placed with different robot platforms yielded weighted accuracy rates of GRS A and B screws: Excelsius GPS^®^, 98.0%; ROSA^®^, 98.0%; Mazor^®^, 98.2%; and Cirq^®^, 94.2%, with no reoperations for ROSA^®^ and 0.28% reoperation rates for Cirq^®^ [24]. In this meta-analysis, Cirq^®^ had the lowest radiation exposure of all platforms [24]. One of the main advantages of RG technology is that the use of low-dose protocols for intraoperative imaging minimizes radiation exposure for staff. In addition, control iCT scans allow the direct control of implant position and reduce the need for postoperative CT. Even without iCT, Gabrovsky et al. [7,25] demonstrated, in their study with a passive-assist robotic arm, that radiation exposure for the surgeon was minimal when using Cirq^®^, although there was no difference in intraoperative radiation exposure compared to the FG group. In their study comparing109 RG and 108 FG screws in 24 and 20 patients, respectively, the rate of GRS A screws was higher in the robotic group (95.4% vs. 88.8%), although the overall accuracy was not different. The operation time and time per screw were significantly lower in the RG group than in the FG group [25]. Desai et al. [26] also used the passive assistance version of Cirq^®^, with 40% of 166 screws placed in RG technique and no misplacements but a longer operating times compared to the FG technique. Interestingly, the same group found an increase in hospital readmissions due to pain, urinary retention, and wound drainage compared to the FG group, all of which were most likely attributed by the authors to the prolonged anesthesia time [26].

As noted by Desai et al. [26], Cirq has a lighter design than floor-mounted platforms that are designed for maximum rigidity of the robotic arm [26]. Due to its bedside mounting, Cirq^®^’s working range is limited, as it must be positioned to reach the planned surgical levels. This can be optimized by positioning the patient, as the upper thoracic area requires the patient to be positioned further caudally on the operating table to allow the device to reach the most cranial levels. In particular, the cranial contralateral level [6] can be challenging when positioning the kinematic unit due to its limited reach, as noted by Desai et al.

### 4.3. Pedicle Screw Accuracy Placement and Robotic Time

The study reports 89% GRS A/B screw accuracy (619/688) with a 2.9% revision rate, slightly lower than larger studies. Screw misplacement is attributed to high rates of osteoporosis and possibly higher rates of tumors and infections in our cohort. Some GRS D screws were retained in long constructs, with no loosening observed, which highlights a pragmatic approach in complex cases. One possible reason for this is also the use of navigation for PSs, as inaccuracies in the registration of the screwdriver can lead to the misplacement of the screws. Cirq^®^ users increasingly recommend that after the introduction of the K-wire, the PS should be introduced either without imaging or with a C-arm. While meta-analyses by Perdomo-Pantoya et al. [12] reported 90.5–95.5% accuracy across techniques, the current study’s slightly lower rate aligns with Ringel et al.’s findings (85% RG accuracy vs. 93% freehand) [27], where skiving and registration errors were major pitfalls. Screw skiving has been recognized by Ringel et al. [27] as a con of RG spine surgery. In their cohort, RG intervention showed less accuracy compared to freehand (85% vs. 93%), with a longer operative time. However, most studies favor RG over freehand/FG techniques in thoracic spine and osteoporosis cases [11,28,29]. The study did not measure time per screw directly (unlike the literature reports of 3–7 min), but noted no difference between open/MIS approaches, consistent with larger cohorts [21,30]. The key contrasts of the current study compared to other meta-analyses include the higher misplacement rate, which we believe reflects the study’s high-risk patient profile, whereas the literature typically reports > 95% accuracy in less complex cohorts. Retention of GRS D screws diverges from strict revision protocols in other studies but underscores real-world adaptability in compromised anatomy [27].

In general, a literature review of 35.630 PSs revealed that nerve root lesions and irritation were reported in a mean of 0.18% and 0.19% per PS, respectively, and that 32 out of 5654 patients required revision due to new neurological deficits caused by misplaced screws [31]. Although comparing the RG technique with FG and iCT-based navigation techniques in our department was not the focus of interest in this manuscript, an analysis of our unpublished data showed that the use of intraoperative imaging resulted in improved PS accuracy rates and fewer screw revisions compared to the FG technique, but with no differences in the rate of GRS A and B screws Orin the rate of misplaced screws between the RG technique and the navigated drill guide. These results are consistent with the literature, which reports a lower complication rate, fewer injuries to the proximal facet joint, and lower radiation exposure, with longer operating times for the RG technique compared to the FG technique [32,33,34,35,36].

The overall accuracy rates in a meta-analysis by Perdomo-Pantoja et al. [12] with 51.161 PSs were 95.5%, 93.1%, 91.5%, and 90.5%, via i-CT based navigation, freehand, FG, and RG techniques [12]. Although these results suggest that the RG technique is less accurate than the others, the heterogeneity of the data and method to determine PS accuracy influenced these results. Compared to other techniques, navigation and RG techniques show fewer breaches in the thoracic spine [12]. In experienced hands, the PS implantation technique seems to play only a minor role in patient outcomes. A randomized controlled trial comparing MISS in the RG context and the open FG technique showed reduced radiation exposure and length of stay, but patient outcomes were not affected by the technique [37]. Asada et al. [28] reported longer operating times with shorter hospital stays and reduced blood loss with RG compared to freehand technique, but with similar complication and readmission rate. The superiority of PS placement accuracy in the RG setting compared to FG was also demonstrated using O-arm based RG technique in terms of reduction in medial and anterior breaches [38].

The continuous development of RG technology leads to improved accuracy, as shown by a literature review and meta-analysis comparing older and newer generations of robots [2]. A difference between 99% for the newer and 97% for the older generation was found [2]. However, a major problem with this comparison is that most studies were retrospective, different robot systems were used, and the criteria for defining PS accuracy also differed in the included manuscripts. Apart from the study of Ringel et al. [27], which showed the inferiority of the RG technique compared to freehand and FG techniques, all other studies showed the superiority of RG technology in terms of PS placement accuracy.

The rate of misplaced screws in our study is slightly higher than previously reported, which can be attributed, among other things, to the high rate of osteoporosis and the inclusion of patients with impaired bone substances, such as patients with infection and tumors. Since K-wires were placed using the RG technique and screws were placed using navigation, both procedures have their own sources of error. Therefore, it is increasingly recommended to place PSs over K-wires without navigation, i.e., using fluoroscopy or without any imaging control. In general, the accuracy of PS placement depends on several factors, including the quality of preoperative and intraoperative image data used to perform trajectory planning and screw implantation. The software allows automatic trajectory planning, but the system is not perfect—it is essential to check each trajectory. Since intraoperative screw malposition occurs due to skive or shift [39], the clinical workflow is important to minimize these risks. It is crucial that the reference array is not too far away from the surgical site, but also not too close, to avoid interfering with the kinematic unit of the robotic arm or the screwdriver, as even slight movement can lead to high inaccuracy. Other sources of inaccuracies include suboptimal calibrations of the screws and loosening of the screw from the screwdriver during implantation. The pressure extended on the vertebrae by the instrument holder attached to the kinematic unit needs to be appropriate, as too little pressure can cause the instrument holder to move and drill outside of the pedicle. In this context, the haptic feedback of the robotic arm, i.e., adjustment to the pressure exerted, is important for stabilizing the K-wire insertion process. To maximize navigation accuracy, all K-wires are implanted first, as navigation accuracy decreases with each screw implantation due to movements in the surgical field. Therefore, we recommend implanting the most critical screws first (part of the spinal canal with the spinal cord, i.e., thoracic screws before lumbar screws and screws to be implanted in smaller pedicles compared to those in larger pedicles). In addition to registration inaccuracies due to the movement of the registration array or due to distance from the reference array to the screw entry point of three levels and more [40], other risk factors include suboptimal calibration of instruments due to loosening of the screw from the screwdriver, skiving due to osteoporosis [40], an unusually steep slope, and a low entry point [39]. A recent review of common errors in RG surgery in 13 studies found that registration errors were the cause of 60% failed screws, with 26.8% of the failed screws attributable to skiving [41]. Interference errors were reported in four studies, with 19.5% of the screws failing [41].

Obesity is a recognized factor for screw misplacement [39,40], but in our current study, it showed no correlation with screw misplacement. A recent multicenter study using the novel CUREXO^®^ robot (CuviSpine) in 448 PSs showed lower GRS A rates of 88.8% compared to the literature, with an average entry deviation error of 2.86 mm [42]. Avrumova et al. [9] reported pedicle breach, i.e., a PS misplacement rate of 5.5%, in their analysis of K-wireless RG PS placement, with (2.9%) critical breaches due to soft tissue pressure leading to skiving. A small pedicle diameter with hypoplasia [9] and congenital scoliosis are additional risk factors for screw misplacements among all techniques. Although the rate of screw misplacement is higher in association with osteoporosis, the RG technique demonstrates greater accuracy in PS placement compared to the freehand technique and other techniques [29]. A systematic review of RG PS placement in scoliosis has shown higher accuracy and longer operating times but comparable clinical outcomes to the freehand and navigation technique [43]. In all studies, the RG technique has the highest incidence of GRS A PS placement, even compared to augmented reality (AR) screws [44]. A retrospective multicenter study showed that the deviation between actual and planned screws was only 18% and that patient- and screw-related variables, such as BMI, gender, the experience level of the treating surgeon, the level of the spine, and the length of the construct, as well as screw length and diameter, had no influence on PS accuracy [45].

Robot abandonment has also been previously described. In their series of 33 patients, Urakov et al. [13] reported that due to “timed-out software” in one planned case, the robot was not used. In this study, the rate of misplaced screws or screws with a breach was 3.2% (10/301 PS). Asada et al. [46] reported superiority of RG PS placement over navigation in all levels of the lumbar spine but with similar efficacy at S1. Apart from a shorter operating time in the percutaneous group, there were no differences between open and MIS surgery in our cohort, which is consistent with the prospective cohort of 1030 PSs, which showed equal accuracy and safety for both the open and MIS RG technique [47]. In short lumbar fusion, the percutaneous RG technique increases radiation exposure but reduces operating time and intraoperative blood loss [48].

In this study, robotic time is defined as the total time during which robotic arm was in operation. Since other robotic systems enable RG PS implantation, it is possible to measure the time per individual screw. In our setup, where all K-wires were placed using a robotic arm, we were able to assess the total time the robotic arm was running and, through dividing this time by the number of screws, indirectly measure the time per individual K-wire. Therefore, a direct comparison with other studies is not possible, and apart from that, the question arises as to the significance of a parameter such as the time per screw for quality control.

The time per screw has been reported differently in the literature: 3 min [49], 4 min [37], or even 7 min [19]. Urakov reported a time per screw of 3.6 min for the midline approach and 5.7 for the percutaneous approach [13]. Differences in robotic and operating time depend on many factors, such as installation time, software problems during the surgery, surgical steps in addition to PS implantation, and the navigation platform. A meta-analysis of 16.040 PSs showed that the Medtronic (Minneapolis, MN, USA), Stryker (Kalamazoo, MI, USA), and BrainLab platforms had similar accuracy, but the surgical time was significantly shorter with Brainlab, and the risk of pedicle breach was lower with all platforms compared to conventional techniques [50]. One of the recognized advantages of RG spinal surgery is the reduction in radiation exposure. In a review of 38 studies by Caelers et al. [51], the use of C-arm resulted in a higher effective dose for surgeons, a higher absorbed dose for patients, and higher exposure compared to C-arm- and O-arm-based navigation and the RG technique. A systematic review of intraoperative radiation involving 85 studies showed that navigation based on intraoperative imaging has the lowest radiation exposure for the surgeon, although there is a relative increase in radiation exposure for the patient compared to the FG technique [52]. Like other studies, the Cirq^®^ system reduced staff radiation, but intraoperative challenges (e.g., reference array positioning, skiving in osteoporotic bone) mirrored known RG limitations [9,27].

### 4.4. Limitations

The study has several limitations, including mixed cohorts of degenerative, traumatic tumorous and infectious spinal disorders. Nevertheless, the aim of the study was to show that RG surgery is suitable for different pathologies. The surgeries were mainly performed by two surgeons with a strong focus on spine surgery to avoid misinterpretation due to expected higher complications rates in the early stages of training. We did not analyze differences in PS accuracy between different techniques (FG, RG, and iCT-based navigation) in detail. The intention of this study was to analyze the RG technique in detail and to illustrate a learning curve in the context associated with the introduction of RG spine surgery in a single center by surgeons who have no previous experience with the RG technique. The cost-effectiveness of the technique was not analyzed due to the complexity of financial coverage for surgeries in a social health care system, where all procedures are covered by health insurance.

## 5. Conclusions

Although interpretation of the results presented in this study is subject to limitations due to small subgroup analyses and limitations of the robotic arm, such as installation problems and other technical issues, as well as the necessity of use of iCT with the RG technique presented, several observations can be made. The Cirq^®^ Robotic Alignment Module from BrainLab enables placement of PSs in the thoracolumbar spine for various pathologies with good accuracy. Accuracy in RG spine surgery depends on image quality, the accuracy of image data, and the accuracy of planned trajectories—planning software, the registration method (with automatic registration being the best option), clinical workflow (positioning of the reference array, pressure applied to the vertebrae, and haptic feedback), and employment of all K-wire techniques first, followed by the implantation of screws, either using a fluoroscopic or navigated technique. Osteoporosis has been shown to be a risk factor for hardware failure. There is a learning curve in terms of improving the robotic time and time per K-wire.

## Data Availability

The data in this study are available on request from the corresponding author. The data are not publicly available due to privacy restrictions.

## Abbreviations

The following abbreviations are used in this manuscript:

RG	robotic guided
PS	pedicle screw
iCT	intraoperative computer tomography
GRS	Gertzbein Robbins system
FDA	Food and Drug Administration
OR	operative room
3D	three-dimensional
MRI	magnetic resonance imaging
K-wire	Kirschner wire
DXA	dual-energy x-ray absorptiometry
HU	Hounsfield unit
ED	effective dose
